# Influence of Digestion Procedure and Residual Carbon on Manganese, Copper, and Zinc Determination in Herbal Matrices by Atomic Absorption Spectrometry

**DOI:** 10.1155/2017/6947376

**Published:** 2017-10-19

**Authors:** Dorota Adamczyk-Szabela, Piotr Anielak, Wojciech M. Wolf

**Affiliations:** Institute of General and Ecological Chemistry, Lodz University of Technology, Zeromskiego 116, 90-924 Lodz, Poland

## Abstract

Mineralization to the complete oxidation of sample carbon component does not always assure the best analyte recovery. Particular attention should be paid to the presence of silicon in the investigated plant sample and especially in the certified reference material for which Si content is scarcely given by the providers. During mineralization without addition of the hydrofluoric acid, the residual carbon may block silica surfaces and increase availability of an analyte for its spectral determination in the solution. This issue is of particular relevance because standard protocols for digestion of plant matrices often do not support hydrofluoric acid addition. Several procedures recommended for decomposition of herbal plants were applied for the respective certified reference material and examined in detail. Manganese, copper, and zinc contents were analyzed in all samples by the flame atomic absorption spectrometry. Additionally, the residual carbon was determined in all mineralizates. Silicon content was analyzed by the X-ray fluorescence method. The best recoveries were observed for samples characterized by relatively high residual carbon.

## 1. Introduction

Accurate determination of heavy metals content in medical herbs and herbal food additives is an important issue in applied analytical chemistry and agriculture [1]. Medicinal plants are widely used all over the world and according to the World Health Organization (WHO) they are the main source of health care for millions of people. Their consumption is growing worldwide in developing and developed countries alike. Herbal therapies are usually long term. Therefore, even small heavy metal doses as present in particular plant may accumulate in patient body over a period of time. This issue prompted numerous works on analytical procedures for metal determination in medical herbs and herbal food supplements. However, papers critically evaluating sample preparation steps and digestion are quite scarce indeed [2].

Modern spectral methods, like AAS or ICP, widely applied for the heavy metal determination in environmental samples require efficient matrix destruction [3, 4]. For years, analytical chemists have been aiming at developing effective methods of mineralization.

Organic or mixed samples are usually brought into solution by some types of oxidation process followed by an acid digestion of the resulting residue. In dry ashing procedures, the organic matter of a sample is decomposed at high temperatures and resulting ash is subsequently dissolved in a strong acid [5]. Usually, mineralization is performed at atmospheric pressure in a programmable furnace at temperatures approaching the range 450–600°C [6]. The important practical advantage of this method is that it allows processing relatively large samples. The resulting ash can be easily dissolved in a small volume of acid enabling efficient preconcentration of trace elements in the final solution [7]. As compared to dry ashing, the wet digestion applies significantly wider range of reagents and methodologies [8]. It may be carried out in open or closed systems, the latter being especially recommended for trace analysis. The breakthrough in modern mineralization techniques came with introduction of microwave energy. The first effective system was developed by Abu-Samra et al. and further applied to biological matrices [9]. Nowadays, this rapid and efficient method is usually realized in commercial, automated closed systems [10, 11].

In wet digestion methods mixture of oxidizing agents (HNO_3_, concentrated HClO_4_, concentrated H_2_SO_4_, or H_2_O_2_) combined with the nonoxidizing mineral acids (HCl, HF, H_3_PO_4_, diluted H_2_SO_4_, and diluted HClO_4_) usually reacts with the analytical sample components [10]. The most abundant oxidizing reagent used for mineralization of complex biological matrices is HNO_3_. However, its oxidizing power is not always sufficient to ensure complete transformation of the sample carbon component to carbon dioxide [12]. For this reason it is often augmented with auxiliary oxidizing regents like HClO_4_ [13, 14], H_2_O_2_ [15], and H_2_SO_4_ [16].

The quality of analytical process should be controlled by parallel experiments with the certified reference materials (CRM). There are numerous data on metal recoveries for plant matrices. Selected digestion methods and metal recoveries reported for manganese, zinc, and copper as determined by AAS or ICP at concentrations commonly observed in plant matrices are summarized in Table 1. Unfortunately, we could hardly find obvious correlations between the determined metal concentrations and reported recovery values. The latter vary significantly and often depend on the digestion procedure [27]. Exceptionally, automatic microwave digestion systems do not necessarily assure the best results when applied to plant samples. Moreover, there is a general understanding that mineralization is crucial to ensure the high quality of an analytical process and careful optimization of all steps involved is of particular relevance. This issue was further investigated by Walas et al. [28] and Blicharska et al. [29]. In particular, the latter analyzed microwave mineralization protocols for herbal samples using the principal component analysis (PCA) and firmly concluded that all mineralization parameters should be carefully optimized prior to the final digestion. In particular, increasing microwave energy and extension of reaction time does not always lead to better heavy metal recoveries. During mineralization, volatile forms of metals could be generated resulting in the systematic loss of an analyte [30]. Therefore, the analyst has to resolve a contradiction (at least when mineralization conditions are concerned) between the organic carbon oxidation requirements and generation of volatile analyte forms. Our observations, supported by Simeonov et al. [31] and Kebbekus [32], clearly indicate that for plant samples the best recoveries are often obtained when digestion combined with limited destruction of the organic matrix components is applied. The latter can be conveniently examined with the total organic carbon (TOC) parameter. This study follows our experience with chemical analysis of plants and investigations of heavy metal migration in herbs [33–35].

Herbs are extensively cultivated worldwide as either important spice and food additives or natural medicines. They are also applied as protective plants and a source of natural biocides to be used in contemporary, sustainable agriculture [36, 37]. Nowadays their production is continually increasing due to a highly efficient farming which employs modern agricultural technologies. Herbs are cultivated on diverse soils and the possibility of heavy metal uptake by these plants cannot be ruled out. Therefore, the accurate determination of heavy metals in herbal plants is an important issue for environmental analytical chemistry and prompted numerous works on relevant laboratory procedures. However, papers critically evaluating sample preparation and digestion are quite scarce indeed [4, 6].

## 2. Materials and Method

The high quality certified reference material (CRM) INCT-MPH-2 containing representative, carefully selected mixture of Polish herbs [38] delivered by the Department of Analytical Chemistry, Institute of Nuclear Chemistry and Technology (Poland), was used throughout this work. Heavy metals concentrations were determined by the flame atomic absorption spectrometry (FAAS). Fifteen widely accepted mineralization methods were selected after the extensive literature and analytical standards survey. They include dry ashing, wet digestion, and microwave techniques frequently used in environmental analytical chemistry.

### 2.1. Method I

All microwave-assisted digestions were performed in a closed system. A 500 mg samples of the CRM were placed into vessels suitable for pressure mineralization in a microwave oven. Next, 6 mL of HNO_3_ (65%) and 1 mL of HCl (36%) were added to each vessel. To explore efficiency of the method seven modifications of temperature and pressure as well as heating and holding times were applied. Respective values are summarized in Table 2.

### 2.2. Method II

500 mg samples of CRM were weighed into Pyrex beakers and treated with 10 mL of HNO_3_ (65%). The beakers were covered with a watch-glass and were heated to boiling on a hot plate. Solutions were maintained at this condition for 40 min and then evaporated to dryness. 5 mL of H_2_O_2_ (30%) were added to the dry residues. Suspensions were heated to boiling and maintained at this temperature for 10 min. After cooling, 10 mL of HNO_3_ (65%) was added and, next, was boiled for 20 min and then evaporated to dryness. Suspensions were soluble in 5 mL of HCl (1 : 1) and were transferred to a 50 mL flask and diluted with distilled water up to the mark. The solutions were then passed through medium filters to plastic bottles.

### 2.3. Method III

10 mL of HNO_3_ (65%) was added to 1000 mg samples of the CRM and suspensions were heated for 45 min at 90°C. The sides of beakers were occasionally washed down with distilled water. The temperature was increased to 140°C and the digestion continued at this temperature until about 1 mL of acid remained. After cooling, the suspensions were transferred to 50 mL flasks and diluted with distilled water up to the mark. The solutions were then passed through medium filters to plastic bottles [13].

### 2.4. Method IV

1000 mg samples of CRM were weighed into Pyrex beakers and treated with 10 mL of HNO_3_ (65%). The beakers were covered with a watch-glass and the suspensions were heated up to 130°C for 1 hour. 4 mL of H_2_O_2_ (20%) was added in aliquots of 1 mL. After cooling, the suspensions were transferred to 50 mL flasks and diluted with distilled water up to the mark. The solutions were then passed through medium filters to plastic bottles [13].

### 2.5. Method V

10 mL of HNO_3_ (65%) was added to 1000 mg samples of CRM and allowed to stand overnight at room temperature. Next, the samples were heated on the hot plate until the reddish-brown NO_2_ fumes disappeared. After cooling, 3 mL of HClO_4_ (70%) was added to the residues and heated again until about 2 mL of acid remained. Next, the suspensions were transferred to 50 mL flasks and diluted with distilled water up to the mark. The solutions were then passed through medium filters to plastic bottles.

### 2.6. Method VI

A 500 mg samples of CRM were weighed into a quartz crucibles, treated with 4 mL of HNO_3_ (65%) and 1 mL of HClO_4_ (70%). The samples were evaporated to dryness. After cooling, HNO_3_ (1 mol L^−1^) was added to residuals. The solutions were transferred to 25 mL flasks, diluted with 1 mol L^−1^ HNO_3_ up to the mark, and finally passed through medium filters to plastic bottles [39].

### 2.7. Method VII

500 mg samples of CRM were weighed into porcelain crucibles. They were ashed in a muffle furnace at 525°C temperature for 7 h. After cooling, the residuals were wetted with distilled water. Next, 2 mL of HNO_3_ (65%) was added and mixtures were evaporated to dryness on a hot plate. Samples were heated again for 1 hour at the temperature 520°C. After cooling, 5 mL of HCl (1 : 1) was added to residuals, transferred to 50 mL flasks, and diluted with distilled water up to the mark. The solutions were then passed through medium filters to plastic bottles [40].

### 2.8. Method VIII

1000 mg samples of the CRM were weighed into porcelain crucibles. They were ashed in a muffle furnace at 500°C temperature for 4 hours. After cooling, the contents of the crucibles were quantitatively transferred to platinum evaporating dishes. Next, 2 mL of HNO_3_ (65%) and 2 mL of HF (40%) were added. The samples were evaporated to dryness on a water bath. After cooling, 1 mL of HNO_3_ (65%) and 1 mL of HF (40%) were added to the residual. This operation was repeated twice. The dry residues were dissolved in 3 mL of HNO_3_ (65%) and then heated on a hot plate to boiling. The contents of the dishes were transferred to 50 mL flasks and diluted with distilled water up to the mark. The solutions were then passed through medium filters to plastic bottles [2].

### 2.9. Method IX

2000 mg samples of the CRM were weighed into porcelain crucibles. They were ashed in a muffle furnace at 500°C temperature for 6 h. After cooling, the contents of crucibles were quantitatively transferred into platinum evaporating dishes. 1 mL of H_2_SO_4_ (98%) and 20 mL of HF (40%) were added. Samples were heated on a hot plate until white fumes SO_3_ appear. After cooling, 15 mL of HF (40%) was added to all residues. The contents of dishes were evaporated to dryness and the heating continued until complete disappearance of white SO_3_ fumes. After cooling, 10 mL of HCl (1 : 1) and 10 mL of distilled water were added. Dishes were covered with watch-glasses and heated in a water bath for about 40 min to dissolve the dry residues. The resulting solutions were then passed through medium filters to 100 mL flasks and diluted with 1% HCl up to the mark.

### 2.10. Metals Determination

The manganese, zinc, and copper content were determined in all mineralizates by atomic absorption spectrometer using an air/acetylene flame. The operating parameters for all metals were close to those recommended by the manufacturer. Analyses were carried out at the most sensitive analytical spectral lines, that is, Mn 279.5 nm, Cu 324.8 nm, and Zn 213.9 nm. Calibration lines were determined for each metal at concentrations ranging from 0.00 to 5.00 *μ*g mL^−1^.

### 2.11. Total Organic Carbon Determination

Residual carbon in digested samples can be determined by several methods including NMR [18] and ICP-OES [21, 27]. Our approach used an established methodology widely applied to wastes and wastewater. The combustion catalytic oxidation method coupled with nondispersive infrared (NDIR) carbon dioxide detection was applied. The nonpurgeable total organic carbon (NPOC) was determined in all mineralizates. In strongly acidic conditions as applied in digestion procedures the NPOC is equivalent to either the residual carbon or the total organic carbon (TOC) [41, 42]. Chemically aggressive combustion products (i.e., halogenated and sulfur compounds) were removed in traps which were filled with copper and brass wool. The Hach Lange IL 550 TOC-TN analyzer was applied.

### 2.12. X-Ray Fluorescence Analysis

The silicon content was determined by X-ray fluorescence analysis for the certified reference material fused to a glassy pellet. The fluorescence intensity was measured with the Thermo Scientific™ ARL™ PERFORM'X sequential X-ray fluorescence WDXRF spectrometer.

### 2.13. Data Analysis

All analyses were replicated six times. The initial assumptions on the equal variances of investigated populations were validated with the Bartlett and Hartley tests as implemented in STATISTICA 10 PL package. Normality of sample distributions was further confirmed with the Shapiro-Wilk test [43, 44]. Student's *t*-test was used to check the null hypothesis whether mean concentrations of metals as determined experimentally were equal to values reported in the CRM. The *α* = 0.05 significance level was applied for all calculations.

The one-way analysis of variance (ANOVA) for manganese, zinc, and cooper contents as compared for all pairs of investigated methodologies was applied.

## 3. Results and Discussion

To our knowledge, this is the most comprehensive, critical evaluation of mineralization procedures as applied in environmental analytical chemistry. All typical approaches including wet, dry, and microwave mineralization were investigated. Fifteen procedures recommended for decomposition of plant matrices were applied for the herbal CRM and examined in detail. Seven used microwave mineralization in a closed system (Ia–Ig), and five applied wet digestion in open arrangement (II–VI), while three were based on dry ashing (VII–IX). Results of analyses expressed as metal contents accompanied by standard uncertainties are summarized in Table 3.

All methodologies were evaluated according to recoveries based on experimental results and metal concentrations as reported in the CRM documentation. Additionally, the NPOC was considered as an important indicator of a matrix organic carbon component oxidation.

Student's *t*-test (Table S1, in Supplementary Material available online at https://doi.org/10.1155/2017/6947376) showed that mean concentrations of metals were not always representative of values reported in the CRM. The best results with recoveries in the range 100 ± 5–103 ± 3% were obtained for the laborious and exceptionally used dry ashing methodology (VII). This method was also featured by the low NPOC levels (below 6 mg L^−1^). On the contrary, wet digestion in an open system (II–VI) was the less reliable methodology giving divergent results. Parameters applied in this procedure should be separately optimized for each metal in the matrix.

**Figure 1:**
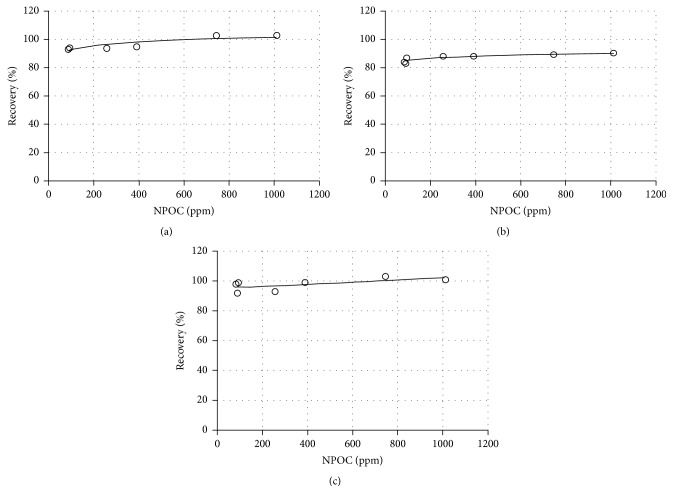
Recoveries versus NPOC as determined for all protocols which were applied for microwave digestion in a closed system: (a) Mn, (b) Zn, and (c) Cu.

The microwave digestion (Ia–Ig) led to a more complicated pattern of recoveries. In this case, quality of analytical results depends strongly on mineralization parameters applied. In general, the If and Ig methods yielded the best recoveries for all metals investigated. Method Ie gave slightly worse results. In all these three approaches the relatively high NPOC values were observed. The lowest metal recoveries were determined for methods Ic and Id and they were associated with the low NPOC values. Similar results were obtained with approach Ia. In particular, method Ib, frequently used for herbal matrices, delivered efficient organic component destruction as confirmed by the low total organic carbon level. Unfortunately, only modest metal recoveries were obtained. In general, the best results were observed for samples characterized by relatively high residual carbon as exemplified by the NPOC values (Figure 1).

**Figure 2:**
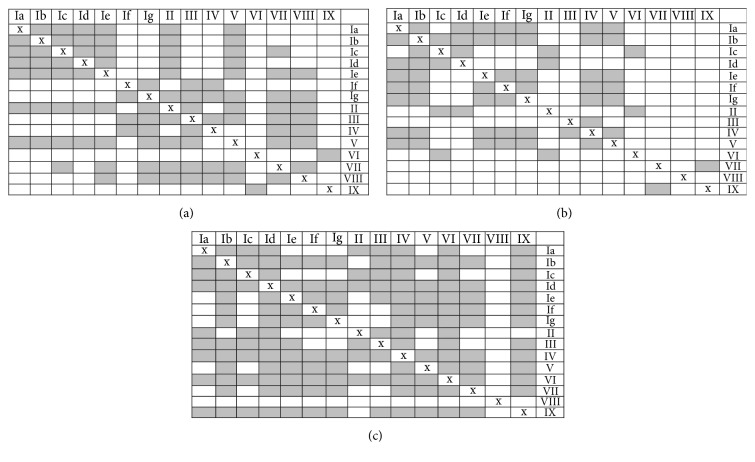
Results of the one-way ANOVA calculations for manganese (a); zinc (b); copper (c) content as compared for all pairs of investigated methodologies. Grey squares represent combinations for which average concentrations are equal at the 0.95 probability level. Numerical values are given in the supplementary materials.

Metals content consistency was controlled by the analysis of variance. The null hypothesis was whether investigated digestion methods led to statistically comparable concentrations. ANOVA calculations clearly showed that they were significantly different when compared across all fifteen methods investigated (Table 4). Similar results were also obtained for computations on smaller datasets representing dry, open wet, and microwave digestions treated separately (Table S2). This negative outcome prompted us to make more detailed comparisons involving all pairs of methods. Results of these multiple one-way ANOVA calculations are visualized in Figure 2 (detailed numerical data are in Supplementary Material, Figures S1–S3). The emerging picture was not straightforward. The best data consistency was observed for copper determination with wet methods IV, VI, and Id. The first two were in open while the last one was in a close digestion setup. Less homogenous results were observed for manganese; the relatively best method II was performed in an open, wet arrangement. On the contrary to dry ashing, only wet methods either in open (IV) or in close (Ib, Ia) systems gave consistent zinc concentrations.

Expanded combined uncertainties and relative standard uncertainties for particular contributions were calculated according to Pauwels et al. [45] for all methods and metal concentrations (Table 5). There is no single method which assures the best precision for all three metals. The lowest expanded combined uncertainty for manganese gives microwave mineralization Ib and wet digestion II. For the zinc and copper altogether the best precision was observed for microwave mineralization Ig while for zinc alone the lowest uncertainty was observed for Id. In all these methods a substantial NPOC contribution was observed.

We would like to stress the importance of silicon derivatives for the quality of the analytical process. This issue was analyzed in combination with the residual carbon as represented by the NPOC results. Among variety of matrix components identified in herbal samples silica is not fully appreciated, nevertheless important factor which influences the final efficiency of digestion procedure. Silicon is the second most abundant element in the Earth's crust, being surpassed only by oxygen. In the form of silicic acid it is readily taken by plants [46].

Matrices with high silicon content (e.g., soils) are mineralized in the presence of hydrofluoric acid which transforms this element to volatile SiF_4_ [47, 48]. However, digestion methods widely applied to plant matrices usually do not use HF [26]. This view is supported by the European Standards [49, 50] and popular microwave protocols. On the contrary, Pöykiö and Perämäki [51] as well as Hoenig and de Kersabiec [8] indicated that HF should always be added for mineralization of plant samples.

Ability to accumulate silicon depends to a great extent either on the character of particular organism or on the cultivation conditions [52, 53]. Therefore, the silicon content in plants can vary significantly, and the values reported in the scientific literature for a shoot dry weight are within the 0.1–10% range [54, 55].

Silicon content in the investigated CRM as determined by the X-ray fluorescence analysis was 0.44 ± 0.03%. Quite similar results were reported by Chuparina and Martynov [56]. It is well recognized that metal ions can absorb on silicon species at a wide range of the pH [57, 58]. We speculate that this phenomenon can be responsible for the decrease of an analyte available for spectral determination. Residual carbon present in sample blocks silica surfaces. Surprisingly, it may be responsible for increase of metals content in the analyte solution and leads to higher recoveries. Less intensive mineralization hinders complete oxidation of the matrix organic carbon component. Its ingredients may further form metaloorganic entities with an analyte. These species if soluble are burnt at high temperature of the AAS furnace or the ICP and finally do not harm accuracy of the metal determination.

## 4. Conclusions

Mineralization of plants samples is frequently regarded as an essentially technical procedure prior to spectral determination of particular metals. In fact, it is a very important step highly responsible for the final quality of the whole analytical process. It should be carefully optimized in relation to either the matrix or the specific analyte. Important factor, which may influence the final metal recovery, is the residual carbon. On the contrary to common expertise, mineralization to the complete oxidation of carbon component in the sample does not always assure the best analyte recovery. Particular attention should be paid to the presence of silicon in the investigated plant sample and especially in the certified reference material. The silicon content is scarcely given by the CRM providers. During commonly used mineralizations without addition of the hydrofluoric acid, the residual carbon blocks silica surfaces and increases availability of an analyte for its spectral determination in the solution.

## Supplementary Material

Figures S1, S2 and S3 in Supplementary Material files show the detailed numerical data for Figure 2. Table S1 presents Student's values calculated for experimentally determined metal concentrations and errors reported in the CRM tabulated for all investigated decomposition methods.

## Figures and Tables

**Table 1:** Selected digestion methods and metal recoveries reported for manganese, zinc, and copper as determined by AAS or ICP at concentrations commonly observed in plant matrices.

Digestion method	Reagents	Detection technique	Reported recoveries in (%)^*∗*^			References
			*Mn*	*Zn*	*Cu*	
Microwave-assisted digestion	HNO_3_/H_2_O_2_	ICP-OES	*95* [72.1 ± 2.5]	*99* [80.9 ± 2.0]	*97* [11.8 ± 1.0]	Araújo et al. [17]

Microwave-assisted digestion	HNO_3_/H_2_O_2_	ICP-OES	*110* [22.1 ± 0.7]	*107* [20.8 ± 0.5]	*94* [2.25 ± 0.13]	Barbosa et al. [18]

Microwave digestion	HNO_3_/HClO_4_/HCl	ICP-AES	*97* [1272 ± 94.6]	*96* [27.3 ± 0.4]	*107* [16.1 ± 2.0]	Başgel and Erdemoğlu [19]

Wet digestion	HNO_3_/H_2_O_2_	AAS	*101* [1590 ± 10]	*108* [37.4 ± 1.0]	*100* [20.5 ± 0.82]	Bielicka-Giełdoń and Ryłko [20]

Microwave digestion	HNO_3_/H_2_O_2_	FAAS	*96* [52.1 ± 3.2]	*102* [12.7 ± 0.8]	*98* [5.53 ± 0.32]	Demirel et al. [2]
Wet digestion	HNO_3_/H_2_O_2_		*95* [51.4 ± 4.9]	*95* [11.9 ± 1.1]	*95* [5.35 ± 0.50]	
Dry ashing	HNO_3_		*94* [50.6 ± 4.7]	*92* [11.5 ± 0.9]	*94* [5.30 ± 0.49]	

Open-vessel, microwave digestion	HNO_3_/H_2_O_2_	ICP-AES	*105* [289 ± 2.66]	*95* [45.9 ± 4.45]	*77* [2.74 ± 0.06]	Huang et al. [21]

Dry ashing	HNO_3_/HF	ICP-AES	*102* [90.5 ± 5.6]	*105* [34.4 ± 2.9]	*97* [7.23 ± 0.3]	Hoenig et al. [4]

Microwave digestion	HNO_3_/H_2_O_2_	ICP-OES	**—**	*89.6* [11.2 ± 2.2]	**—**	Kula et al. [22]

Microwave digestion	HNO_3_/H_2_O_2_/H_2_SO_4_	AAS	*94* [92.0 ± 1.0]	*117* [21.0 ± 0.1]	**—**	Naeem et al. [23]

Dry ashing	HNO_3_	AAS	*97* [67.0 ± 8.0]	*96* [19.7 ± 1.5]	*97* [3.4 ± 0.7]	Tüzen [24]
Wet digestion	HNO_3_/HCl		*99* [68.0 ± 7.0]	*97* [19.9 ± 1.8]	*95* [3.3 ± 0.6]	
Microwave digestion	HNO_3_/H_2_O_2_		*100* [69.0 ± 6.0]	*99* [20.3 ± 1.4]	*103* [3.6 ± 0.5]	

Dry ashing	HNO_3_	FAAS	*97* [65.2 ± 5.4]	*96* [25.4 ± 2.3]	*96* [10.2 ± 1.1]	Soylak et al. [25]
Wet digestion	HNO_3_/H_2_O_2_		*98* [71.4 ± 6.6]	*97* [22.7 ± 1.8]	*97* [11.4 ± 1.0]	
Microwave digestion	HNO_3_/H_2_O_2_		*98* [62.6 ± 3.2]	*100* [27.2 ± 1.3]	*101* [12.8 ± 0.6]	

Microwave-assisted digestion	HNO_3_, HF, HClO_4_, H_2_O_2_	AAS	**—**	*95* [29.8 ± 0.4]	*100* [9.6 ± 0.1]	Sastre et al. [26]

^*∗*^Metal contents in [*µ*g g^−1^] accompanied by their standard deviations as determined in original publications are given in square brackets below the recovery values.

**Table 2:** Parameters modifications as applied to microwave digestion.

Modification number	Ia	Ib	Ic	Id	Ie	If	Ig
Final temperature (°C)	197	211	209	216	178	106	142
Final pressure (bar)	46	60	60	60	37	16	22
Heating time (min)	10	10	10	5	5	5	5
Holding time (min)	5	10	15	15	5	5	5
Cooling time (min)	15	15	15	15	15	15	15

**Table 3:** Metal contents, recoveries, and nonpurgeable organic carbon (NPOC) in certified reference materials containing herbs. All values are accompanied by standard uncertainties. Digestion in the closed microwave system (Ia–Ig); wet digestion in the open system (II–VI); and dry ashing in the open system (VII–IX) followed by the FAAS were applied. Metal concentrations reported for CRM were Mn = 191 ± 12 *µ*g g^−1^; Zn = 33.5 ± 2.1 *µ*g g^−1^; Cu = 7.77 ± 0.53 *µ*g g^−1^.

Method	Metal (*µ*g g^−1^)						NPOC mg L^−1^
	*Mn*	Recovery%	*Zn*	Recovery%	*Cu*	Recovery%	
Ia	179 ± 8	94 ± 4	29.5 ± 1.6	88 ± 5	7.24 ± 0.43	93 ± 5	258 ± 2
Ib	180 ± 6	94 ± 3	29,1 ± 1.3	87 ± 4	7.66 ± 0.40	99 ± 5	94.0 ± 1.5
Ic	179 ± 9	94 ± 5	27.7 ± 1.1	83 ± 3	7.17 ± 0.54	92 ± 7	91.0 ± 1.0
Id	178 ± 8	93 ± 4	28.1 ± 0.8	84 ± 2	7.59 ± 0.44	98 ± 6	84.3 ± 0.8
Ie	181 ± 7	95 ± 4	29.6 ± 1.0	88 ± 3	7.74 ± 0.30	99 ± 4	391 ± 4
If	196 ± 8	103 ± 4	30.0 ± 1.0	90 ± 3	7.82 ± 0.42	101 ± 5	1012 ± 11
Ig	196 ± 9	103 ± 5	29.7± 0.9	89 ± 3	7.98 ± 0.26	103 ± 3	745 ± 7
II	186 ± 6	97 ± 3	26.6 ± 2,1	79 ± 6	7.05 ± 0.48	91 ± 6	275 ± 2
III	197 ± 11	103 ± 6	32.2 ± 1.7	96 ± 5	7.21 ± 0.50	93 ± 6	3140 ± 22
IV	201 ± 7	105 ± 4	30.3 ± 1.4	90 ± 4	7.55 ± 0.60	97 ± 8	1857 ± 13
V	186 ± 7	97 ± 4	30.2 ± 1.1	90 ± 3	7.94 ± 0.37	102 ± 5	558 ± 2
VI	164 ± 7	86 ± 4	26.2 ± 1.5	78 ± 4	7.61 ± 0.45	98 ± 6	3300 ± 22
VII	190 ± 9	100 ± 5	34.4 ± 1.1	103 ± 3	7.93 ± 0.30	102 ± 4	5.62 ± 0.11
VIII	192 ± 9	101 ± 5	40.5 ± 1.1	121 ± 3	9.64 ± 0.50	124 ± 6	10.5 ± 0.2
IX	166 ± 10	87 ± 5	35.2 ± 0.9	105 ± 3	7.68 ± 0.39	99 ± 5	6.25 ± 0.12

**Table 4:** ANOVA parameters for metals content in the CRM across all fifteen investigated methods of digestion.

Source of variation	SS	MS	*F*	*p* value	Test *F*
Zinc	1148.705	82.050	48.208	1.13·10^−31^	1.8259
Copper	30.872	2.205	11.534	1.42·10^−13^	1.8259
Manganese	11735.6	838.257	11.974	5.80·10^−14^	1.8259

**Table 5:** Expanded combined uncertainties and relative standard uncertainties for particular contributions calculated for all methods and metal concentrations.

Method	Relative standard uncertainty				Expanded combined uncertainty *µ*g g^−1^		
	CRM mass sample	Volumetric flask	Pipette^a^	Standard solutions concentration^b^	*Mn*	*Zn*	*Cu*
Ia	0.0002	0.0016	0.0005	0.002	20.4	4.11	1.11
Ib					15.4	3.34	1.03
Ic					23.1	2.83	1.39
Id					20.6	2.06	1.13
Ie					18.0	2.57	0.77
If					21.2	2.57	1.08
Ig					23.1	2.31	0.67

II	0.0002	0.0012	0.0005	0.002	15.4	5.40	1.23
III	0.0001				28.3	4.37	1.29
IV					18.0	3.60	1.54
V					18.0	2.83	0.95

VI	0.0002	0.0016	0.0005	0.002	18.0	3.86	1.16
VII		0.0012			23.1	2.83	0.77
VIII	0.0001				23.1	2.83	1.29
IX	0.00005	0.001			25.7	2.31	1.00

^a^Pipette volume *V* = 10 mL; ^b^concentrations of all Mn, Zn, and Cu standard solutions were *c* = 1.0000 mg L^−1^.
