# Supplementary material for: Influence of Digestion Procedure and Residual Carbon on Manganese, Copper, and Zinc Determination in Herbal Matrices by Atomic Absorption Spectrometry
Source: J Anal Methods Chem. 2017 Oct 19;2017:6947376. doi: 10.1155/2017/6947376 (PMC5662835; doi:10.1155/2017/6947376)
Supplement: Supplementary file 1 — Figures S1, S2 and S3 in Supplementary Material files show the detailed numerical data for Figure 2. Table S1 presents Student's values calculated for experimentally determined metal concentrations and errors reported in the CRM tabulated for all investigated decomposition methods. [file 6947376.f1.pdf]

Figure S1. Results of the one-way ANOVA calculations for manganese content as compared for all pairs of investigated methodologies at the 0.95 probability level.

Ib	Ic	Id	Ie	If	Ig	II	III	IV	V	VI	VII	VIII	IX	
F=0.026076<br>$p=874.9\cdot10^{-3}$	F=0.00114<br>$p=973.7\cdot10^{-3}$	F=0.047022<br>$p=832.7\cdot10^{-3}$	F=0.218182<br>$p=650.4\cdot10^{-3}$	F=30.76184<br>$p=0.245\cdot10^{-3}$	F=11.15076<br>$P=7.50\cdot10^{-3}$	F=2.893701<br>$p=119.8\cdot10^{-3}$	F=10.27484<br>$p=9.41\cdot10^{-3}$	F=25.74468<br>$p=0.482\cdot10^{-3}$	F=2.518892<br>$p=143.6\cdot10^{-3}$	F=11.6451<br>$p=6.63\cdot10^{-3}$	F=5.008666<br>$p=49.17\cdot10^{-3}$	F=6.272806<br>$p=31.20\cdot10^{-3}$	F=6.655574<br>$p=27.42\cdot10^{-3}$	Ia
	F=0.033485<br>$p=858.4\cdot10^{-3}$	F=0.157431<br>$p=699.9\cdot10^{-3}$	F=0.120846<br>$p=735.3\cdot10^{-3}$	F=35.04611<br>$p=0.147\cdot10^{-3}$	F=12.02505<br>$p=6.044\cdot10^{-3}$	F=3.013356<br>$p=113.2\cdot10^{-3}$	F=10.76433<br>$p=8.28\cdot10^{-3}$	F=29.98536<br>$p=0.271\cdot10^{-3}$	F=2.567353<br>$p=140.2\cdot10^{-3}$	F=15.73117<br>$p=2.658\cdot10^{-3}$	F=5.166498<br>$p=46.33\cdot10^{-3}$	F=6.558704<br>$p=28.32\cdot10^{-3}$	F=8.488932<br>$p=15.47\cdot10^{-3}$	Ib
		F=0.027821<br>$p=870.9\cdot10^{-3}$	F=0.213115<br>$p=654.2\cdot10^{-3}$	F=26.62582<br>$p=0.425\cdot10^{-3}$	F=9.905807<br>$p=10.38\cdot10^{-3}$	F=2.489900<br>$p=145.7\cdot10^{-3}$	F=9.368396<br>$p=12.03\cdot10^{-3}$	F=21.84367<br>$p=0.876\cdot10^{-3}$	F=2.188232<br>$p=169.9\cdot10^{-3}$	F=9.522873<br>$p=11.52\cdot10^{-3}$	F=4.507669<br>$p=59.71\cdot10^{-3}$	F=5.626305<br>$p=39.14\cdot10^{-3}$	F=5.701626<br>$p=38.10\cdot10^{-3}$	Ic
			F=0.475352<br>$p=506.2\cdot10^{-3}$	F=32.29629<br>$p=0.203\cdot10^{-3}$	F=12.23432<br>$P=5.748\cdot10^{-3}$	F=3.650190<br>$p=85.13\cdot10^{-3}$	F=11.23444<br>$p=7.34\cdot10^{-3}$	F=27.26804<br>$p=0.389\cdot10^{-3}$	F=3.221681<br>$p=102.9\cdot10^{-3}$	F=9.816187<br>$p=10.64\cdot10^{-3}$	F=5.795936<br>$p=36.84\cdot10^{-3}$	F=7.134975<br>$p=23.44\cdot10^{-3}$	F=5.569569<br>$p=39.95\cdot10^{-3}$	Id
				F=29.33859<br>$p=0.294\cdot10^{-3}$	F=9.527559<br>$p=11.51\cdot10^{-3}$	F=1.712329<br>$p=219.9\cdot10^{-3}$	F=8.767123<br>$p=14.27\cdot10^{-3}$	F=24.2915<br>$p=0.597\cdot10^{-3}$	F=1.422351<br>$p=260.5\cdot10^{-3}$	F=17.11003<br>$p=2.023\cdot10^{-3}$	F=3.736892<br>$p=82.01\cdot10^{-3}$	F=4.894837<br>$p=51.34\cdot10^{-3}$	F=9.644485<br>$p=11.15\cdot10^{-3}$	Ie
					F=3.239452<br>$p=102.1\cdot10^{-3}$	F=19.74791<br>$p=1.247\cdot10^{-3}$	F=1.884491<br>$p=199.8\cdot10^{-3}$	F=0.741104<br>$p=409.5\cdot10^{-3}$	F=19.6742<br>$p=1.26\cdot10^{-3}$	F=83.01171<br>$p=0.004\cdot10^{-3}$	F=7.882733<br>$p=18.55\cdot10^{-3}$	F=6.515974<br>$p=28.73\cdot10^{-3}$	F=55.30242<br>$p=0.022\cdot10^{-3}$	If
						F=4.412382<br>$p=62.02\cdot10^{-3}$	F=0.049689<br>$p=828.1\cdot10^{-3}$	F=1.23433<br>$p=292.6\cdot10^{-3}$	F=4.568528<br>$p=58.29\cdot10^{-3}$	F=42.88235<br>$p=0.065\cdot10^{-3}$	F=0.951673<br>$p=352.3\cdot10^{-3}$	F=0.536513<br>$P=480.7\cdot10^{-3}$	F=29.07394<br>$p=0.305\cdot10^{-3}$	Ig
							F=4.352518<br>$p=63.53\cdot10^{-3}$	F=14.93363<br>$p=3.14\cdot10^{-3}$	F=0.007987<br>$p=930.5\cdot10^{-3}$	F=31.44192<br>$p=0.225\cdot10^{-3}$	F=0.856998<br>$p=376.4\cdot10^{-3}$	F=1.469990<br>$p=253.2\cdot10^{-3}$	F=17.99323<br>$p=1.711\cdot10^{-3}$	II
								F=0.539326<br>$p=479.6\cdot10^{-3}$	F=4.505066<br>$p=59.77\cdot10^{-3}$	F=36.2227<br>$p=0.129\cdot10^{-3}$	F=1.217285<br>$p=295.7\cdot10^{-3}$	F=0.780488<br>$p=397.7\cdot10^{-3}$	F=26.12655<br>$p=0.456\cdot10^{-3}$	III
									F=14.90141<br>$p=3.16\cdot10^{-3}$	F=79.67537<br>$p=0.004\cdot10^{-3}$	F=4.785047<br>$p=53.56\cdot10^{-3}$	F=3.673852<br>$p=84.26\cdot10^{-3}$	F=50.25045<br>$p=0.033\cdot10^{-3}$	IV
										F=29.12321<br>$p=0.303\cdot10^{-3}$	F=0.962672<br>$p=349.7\cdot10^{-3}$	F=1.596059<br>$p=235.1\cdot10^{-3}$	F=16.88555<br>$p=2.113\cdot10^{-3}$	V
											F=28.68164<br>$p=0.321\cdot10^{-3}$	F=31.76779<br>$p=0.217\cdot10^{-3}$	F=0.090221<br>$p=770.0\cdot10^{-3}$	VI
												F=0.058182<br>$p=814.3\cdot10^{-3}$	F=19.200565<br>$p=1.373\cdot10^{-3}$	VII
													F=21.37737<br>$p=0.945\cdot10^{-3}$	VIII

Figure S2. Results of the one-way ANOVA calculations for zinc content as compared for all pairs of investigated methodologies at the 0.95 probability level.

Ib	Ic	Id	Ie	If	Ig	II	III	IV	V	VI	VII	VIII	IX	
F=0.35433<br>$p=564.9\cdot10^{-3}$	F=5.91587<br>$p=35.31\cdot10^{-3}$	F=3.80865<br>$p=79.54\cdot10^{-3}$	F=0.01753<br>$p=897.2\cdot10^{-3}$	F=0.32388<br>$p=581.8\cdot10^{-3}$	F=0.050731<br>$p=826.3\cdot10^{-3}$	F=7.424736<br>$p=21.38\cdot10^{-3}$	F=7.681209<br>$p=19.74\cdot10^{-3}$	F=0.658996<br>$p=435.8\cdot10^{-3}$	F=0.615281<br>$p=451.0\cdot10^{-3}$	F=13.90821<br>$p=3.914\cdot10^{-3}$	F=38.02355<br>$p=0.106\cdot10^{-3}$	F=187.2329<br>$p=0.00008\cdot10^{-3}$	F=56.55644<br>$p=0.0202\cdot10^{-3}$	Ia
	F=3.939259<br>$p=75.28\cdot10^{-3}$	F=2.028296<br>$p=184.8\cdot10^{-3}$	F=0.78679<br>$p=395.9\cdot10^{-3}$	F=1.864891<br>$p=202.0\cdot10^{-3}$	F=1.021972<br>$p=335.9\cdot10^{-3}$	F=5.659516<br>$p=38.68\cdot10^{-3}$	F=12.47378<br>$p=5.43\cdot10^{-3}$	F=2.282494<br>$p=161.8\cdot10^{-3}$	F=2.467297<br>$p=147.3\cdot10^{-3}$	F=11.69851<br>$p=6.544\cdot10^{-3}$	F=56.44817<br>$p=0.020\cdot10^{-3}$	F=248.6243<br>$p=0.00002\cdot10^{-3}$	F=83.88503<br>$p=0.0035\cdot10^{-3}$	Ib
		F=0.732451<br>$p=412.1\cdot10^{-3}$	F=11.04294<br>$p=7.707\cdot10^{-3}$	F=14.64435<br>$p=3.336\cdot10^{-3}$	F=12.57853<br>$p=5.298\cdot10^{-3}$	F=1.122926<br>$p=314.2\cdot10^{-3}$	F=29.85164<br>$p=0.276\cdot10^{-3}$	F=12.62241<br>$p=5.244\cdot10^{-3}$	F=15.44574<br>$p=2.820\cdot10^{-3}$	F=3.476871<br>$p=91.81\cdot10^{-3}$	F=110.3615<br>$p=0.001\cdot10^{-3}$	F=382.6149<br>$p=0.000\cdot10^{-3}$	F=160.7174<br>$p=0.000\cdot10^{-3}$	Ic
			F=8.246365<br>$p=16.622\cdot10^{-3}$	F=11.86308<br>$p=6.286\cdot10^{-3}$	F=9.782137<br>$p=10.733\cdot10^{-3}$	F=2.651277<br>$p=134.5\cdot10^{-3}$	F=27.10794<br>$p=0.398\cdot10^{-3}$	F=9.906031<br>$p=10.38\cdot10^{-3}$	F=12.65216<br>$p=5.207\cdot10^{-3}$	F=7.237058<br>$p=22.69\cdot10^{-3}$	F=119.056<br>$p=0.0007\cdot10^{-3}$	F=439.3351<br>$p=0.0000\cdot10^{-3}$	F=184.197<br>$p=0.000\cdot10^{-3}$	Id
				F=0.334896<br>$p=575.6\cdot10^{-3}$	F=0.014837<br>$p=905.5\cdot10^{-3}$	F=10.10124<br>$p=9.849\cdot10^{-3}$	F=9.844141<br>$p=10.55\cdot10^{-3}$	F=0.726457<br>$p=413.9\cdot10^{-3}$	F=0.721363<br>$p=415.6\cdot10^{-3}$	F=21.39774<br>$p=0.942\cdot10^{-3}$	F=60.60759<br>$p=0.0015\cdot10^{-3}$	F=300.5834<br>$p=0.000\cdot10^{-3}$	F=97.78566<br>$p=0.0017\cdot10^{-3}$	Ie
					F=0.23005<br>$p=641.8\cdot10^{-3}$	F=12.3141<br>$p=5.640\cdot10^{-3}$	F=7.300689<br>$p=22.24\cdot10^{-3}$	F=0.140783<br>$p=715.3\cdot10^{-3}$	F=0.088541<br>$p=772.1\cdot10^{-3}$	F=25.31523<br>$p=0.513\cdot10^{-3}$	F=51.12428<br>$p=0.031\cdot10^{-3}$	F=275.625<br>$p=0.0000\cdot10^{-3}$	F=83.80165<br>$p=0.0036\cdot10^{-3}$	If
						F=10.81936<br>$p=8.160\cdot10^{-3}$	F=9.670187<br>$p=11.07\cdot10^{-3}$	F=0.601504<br>$p=455.9\cdot10^{-3}$	F=0.58292<br>$p=462.8\cdot10^{-3}$	F=23.19100<br>$p=0.707\cdot10^{-3}$	F=62.71609<br>$p=0.0129\cdot10^{-3}$	F=315.1934<br>$p=0.0000\cdot10^{-3}$	F=102.8921<br>$p=0.0014\cdot10^{-3}$	Ig
							F=25.0009<br>$p=0.537\cdot10^{-3}$	F=12.17565<br>$p=5.829\cdot10^{-3}$	F=13.1451<br>$p=4.646\cdot10^{-3}$	F=0.140406<br>$p=715.7\cdot10^{-3}$	F=63.14114<br>$p=0.013\cdot10^{-3}$	F=1966.3867<br>$p=0.00007\cdot10^{-3}$	F=81.38122<br>$p=0.004\cdot10^{-3}$	II
								F=4.497993<br>$p=59.94\cdot10^{-3}$	F=5.786851<br>$p=36.961\cdot10^{-3}$	F=40.59661<br>$p=0.081\cdot10^{-3}$	F=7.273126<br>$p=22.43\cdot10^{-3}$	F=97.19767<br>$p=0.181\cdot10^{-3}$	F=14.34746<br>$p=3.556\cdot10^{-3}$	III
									F=0.01278<br>$p=912.2\cdot10^{-3}$	F=22.46241<br>$p=0.793\cdot10^{-3}$	F=31.66802<br>$p=0.219\cdot10^{-3}$	F=185.7489<br>$p=0.00009\cdot10^{-3}$	F=50.03198<br>$p=0.034\cdot10^{-3}$	IV
										F=26.05966<br>$p=0.461\cdot10^{-3}$	F=42.72901<br>$p=0.066\cdot10^{-3}$	F=242.8451<br>$p=0.00002\cdot10^{-3}$	F=69.72526<br>$p=0.008\cdot10^{-3}$	V
											F=111.4065<br>$p=0.001\cdot10^{-3}$	F=328.7581<br>$p=0.000\cdot10^{-3}$	F=148.4024<br>$p=0.0003\cdot10^{-3}$	VI
												F=83.05918<br>$p=0.0037\cdot10^{-3}$	F=1.606926<br>$p=233.6\cdot10^{-3}$	VII
													F=73.66259<br>$p=0.006\cdot10^{-3}$	VIII

Figure S3. Results of the one-way ANOVA calculations for cooper content as compared for all pairs of investigated methodologies at the 0.95 probability level.

Ib	Ic	Id	Ie	If	Ig	II	III	IV	V	VI	VII	VIII	IX	
F=2.92208<br>p=118.2·10^{-3}	F=0.06741<br>p=800.4·10^{-3}	F=1.87560<br>p=200.8·10^{-3}	F=5.35116<br>p=43.27·10^{-3}	F=5.44383<br>p=41.82·10^{-3}	F=12.4723<br>p=5.432·10^{-3}	F=0.58660<br>p=461.4·10^{-3}	F=0.01794<br>p=896.1·10^{-3}	F=0.95736<br>p=350.9·10^{-3}	F=8.87602<br>p=13.825·10^{-3}	F=2.08373<br>p=179.4·10^{-3}	F=10.2995<br>p=9.343·10^{-3}	F=77.7195<br>p=0.0050·10^{-3}	F=3.21497<br>p=103.2·10^{-3}	Ia
	F=3.13557<br>p=107.0·10^{-3}	F=0.07123<br>p=794.9·10^{-3}	F=0.18595<br>p=675.6·10^{-3}	F=0.47658<br>p=505.7·10^{-3}	F=2.71137<br>p=130.7·10^{-3}	F=5.75466<br>p=37.39·10^{-3}	F=2.86317<br>p=121.5·10^{-3}	F=0.14561<br>p=710.7·10^{-3}	F=1.58480<br>p=236.7·10^{-3}	F=0.03388<br>p=857.6·10^{-3}	F=1.86811<br>p=201.6·10^{-3}	F=56.9251<br>p=0.0196·10^{-3}	F=0.00531<br>p=943.3·10^{-3}	Ib
		F=2.17518<br>p=171.0·10^{-3}	F=5.15697<br>p=46.50·10^{-3}	F=5.40583<br>p=42.41·10^{-3}	F=10.8294<br>p=8.139·10^{-3}	F=0.19321<br>p=669.7·10^{-3}	F=0.01462<br>p=906.2·10^{-3}	F=1.25590<br>p=288.6·10^{-3}	F=8.24609<br>p=16.62·10^{-3}	F=2.37420<br>p=154.3·10^{-3}	F=9.21262<br>p=12.56·10^{-3}	F=67.0212<br>p=0.009·10^{-3}	F=3.39683<br>p=95.12·10^{-3}	Ic
			F=0.48940<br>p=500.1·10^{-3}	F=0.84015<br>p=380.9·10^{-3}	F=3.37849<br>p=95.90·10^{-3}	F=4.21289<br>p=67.22·10^{-3}	F=1.92635<br>p=195.3·10^{-3}	F=0.02478<br>p=878.1·10^{-3}	F=2.14475<br>p=173.8·10^{-3}	F=0.00607<br>p=939.4·10^{-3}	F=2.47914<br>p=146.4·10^{-3}	F=55.7898<br>p=0.0214·10^{-3}	F=0.11422<br>p=742.3·10^{-3}	Id
				F=0.12584<br>p=730.2·10^{-3}	F=2.02434<br>p=185.2·10^{-3}	F=9.08536<br>p=13.02·10^{-3}	F=4.88771<br>p=51.49·10^{-3}	F=0.52863<br>p=483.9·10^{-3}	F=0.95997<br>p=350.3·10^{-3}	F=0.36665<br>p=558.3·10^{-3}	F=1.19567<br>p=299.8·10^{-3}	F=62.1647<br>p=0.0134·10^{-3}	F=0.12517<br>p=730.8·10^{-3}	Ie
					F=0.61992<br>p=449.3·10^{-3}	F=8.84864<br>p=13.93·10^{-3}	F=5.12932<br>p=46.98·10^{-3}	F=0.84162<br>p=380.5·10^{-3}	F=0.25619<br>p=623.7·10^{-3}	F=0.69441<br>p=424.1·10^{-3}	F=0.29916<br>p=596.4·10^{-3}	F=46.0687<br>p=0.048·10^{-3}	F=0.39076<br>p=545.9·10^{-3}	If
						F=17.2496<br>p=1.970·10^{-3}	F=10.72521<br>p=8.360·10^{-3}	F=2.59324<br>p=138.4·10^{-3}	F=0.05921<br>p=812.7·10^{-3}	F=3.00934<br>p=113.4·10^{-3}	F=0.07560<br>p=788.9·10^{-3}	F=50.66227<br>p=0.0323·10^{-3}	F=2.49926<br>p=145.0·10^{-3}	Ig
							F=0.33594<br>p=575.0·10^{-3}	F=2.49929<br>p=144.9·10^{-3}	F=13.0042<br>p=4.799·10^{-3}	F=4.49689<br>p=59.96·10^{-3}	F=14.7991<br>p=3.234·10^{-3}	F=82.7972<br>p=0.0038·10^{-3}	F=6.15782<br>p=32.46·10^{-3}	II
								F=1.067042<br>p=325.9·10^{-3}	F=8.01779<br>p=17.79·10^{-3}	F=2.12019<br>p=176.0·10^{-3}	F=9.03687<br>p=13.20·10^{-3}	F=68.6984<br>p=0.0086·10^{-3}	F=3.12131<br>p=107.7·10^{-3}	III
									F=1.82941<br>p=205.9·10^{-3}	F=0.04929<br>p=828.8·10^{-3}	F=2.00682<br>p=186.9·10^{-3}	F=42.15951<br>p=0.0696·10^{-3}	F=0.19362<br>p=669.3·10^{-3}	IV
										F=1.88682<br>p=199.6·10^{-3}	F=0<br>p=1	F=44.8711<br>p=0.054·10^{-3}	F=1.42782<br>p=259.7·10^{-3}	V
											F=2.17826<br>p=170.8·10^{-3}	F=54.3771<br>p=0.0239·10^{-3}	F=0.06463<br>p=804.4·10^{-3}	VI
												F=50.7205<br>p=0.0321·10^{-3}	F=1.68891<br>p=222.9·10^{-3}	VII
													F=56.7370<br>p=0.020·10^{-3}	VIII

Table S1. Student's t values calculated for experimentally determined metal concentrations and errors reported in the CRM tabulated for all investigated decomposition methods. Values below the critical $t_{6;0.05}$ = 2.571 indicate that null hypothesis (i.e. mean concentration is equal to the metal concentration reported in the CRM) cannot be rejected.

Decomposition method	t value		
	**Mn**	**Zn**	**Cu**
Ia	2.449	4.666	2.449
Ib	2.245	5.132	0.508
Ic	2.449	6.765	2.773
Id	2.654	5.144	0.832
Ie	2.041	4.549	0.139
If	2.041	4.082	0.231
Ig	1.021	4.432	0.971
II	1.021	8.048	3.328
III	1.225	1.516	2.588
IV	2.041	3.733	1.017
V	1.021	3.849	0.786
VI	5.511	8.515	0.739
VII	0.204	1.050	0.739
VIII	0.204	8.165	8.643
IX	5.103	1.983	0.416

Table S2. ANOVA parameters for metals content in the CRM across microwave, open wet and dry methods of digestion.

Decomposition method	Source of variation	SS	MS	F	p-value	Test F
Microwave mineralization (Ia-Ig)	Zinc	28.2291	4.7048	3.6722	$6.21 \cdot 10^{-3}$	2.3718
	Copper	3.1595	0.5266	3.1387	$1.44 \cdot 10^{-2}$	2.3718
	Manganese	4044.2	674.040	10.3546	$1.37 \cdot 10^{-6}$	2.3718
Wet (II-VI)	Zinc	158.0633	39.5158	15.2085	$1.98 \cdot 10^{-6}$	2.7587
	Copper	2.9615	0.7404	3.0898	$3.39 \cdot 10^{-2}$	2.7587
	Manganese	4919.2	1229.8	19.6558	$0.20 \cdot 10^{-6}$	2.7587
Dry (VII-IX)	Zinc	130.9644	65.4822	55.0168	$0.12 \cdot 10^{-6}$	3.6823
	Copper	13.5888	6.7944	41.1491	$0.81 \cdot 10^{-6}$	3.6823
	Manganese	2572.4	1286.2	13.7026	$4.12 \cdot 10^{-4}$	3.6823
